# Comprehensive Evaluation of Quality of Life following Upper Eyelid Blepharoplasty: A Prospective Analysis

**DOI:** 10.3390/medicina60030500

**Published:** 2024-03-19

**Authors:** Dolika D. Vasović, Miodrag Lj. Karamarković, Milan Jovanović, Milan Stojičić, Dejan M. Rašić, Ivan Marjanović, Tanja Kalezić, Miroslav Jeremić

**Affiliations:** 1 University Eye Hospital Clinical Centre of Serbia, 11000 Belgrade, Serbia; 2 Clinic for Burns, Plastic and Reconstructive Surgery, University Clinical Centre of Serbia, 11000 Belgrade, Serbia; 3 Faculty of Medicine, University of Belgrade, 11000 Belgrade, Serbia

**Keywords:** blepharoplasty, quality of life, suturing techniques, patient-reported outcomes, facial aesthetics

## Abstract

*Background and Objectives:* Upper eyelid blepharoplasty is a surgical procedure that addresses both aesthetic and functional concerns, offering transformative potential for patients’ overall well-being. This study systematically evaluates the comprehensive impact of upper eyelid blepharoplasty on patients’ quality of life, employing rigorous methodologies and standardized assessment protocols. *Materials and Methods:* A prospective, randomized controlled trial was conducted, involving 348 patients aged 49 to 87 years. Patients were randomly assigned to receive either continuous or intradermal sutures following upper eyelid surgery. Validated FACE-Q questionnaires were used to assess various outcomes, including early-life impact, expectations, satisfaction with eyes, overall face satisfaction, satisfaction with the outcome, psychological function, social function, and adverse effects. *Results:* Results indicate significant improvements in multiple domains of patient-reported outcomes following upper eyelid blepharoplasty, including satisfaction with eyes, overall face satisfaction, satisfaction with the outcome, psychological function, and social function. Notably, no significant differences were observed between suturing techniques regarding patient satisfaction and well-being. Adverse effects were minimal and improved over time. *Conclusions:* The study underscores the transformative nature of upper eyelid blepharoplasty in enhancing patients’ quality of life, addressing both cosmetic and functional concerns. Utilizing standardized assessment tools like the FACE-Q questionnaire facilitates a comprehensive understanding of treatment outcomes and enables patient-centered care. Overall, this research contributes to the growing evidence supporting the positive impact of upper eyelid blepharoplasty on patients’ well-being, emphasizing the importance of continued research and standardized assessment protocols in advancing patient care in cosmetic surgery.

## 1. Introduction

Upper eyelid blepharoplasty, a meticulous surgical procedure addressing both aesthetic and functional concerns, holds transformative potential for patients’ overall well-being. Through the precise removal of excess skin, adjustment of fat deposits, and anatomical corrections, it not only achieves a rejuvenated appearance but also boosts self-esteem and confidence [1].

The recent scientific literature underscores its psychological impact, as it positively influences self-perceptions and emotional well-being. Studies have shown that patients often experience significant improvements in self-esteem, body image, and overall satisfaction postoperatively, highlighting the procedure’s multifaceted benefits [1]. Moreover, functional improvements, such as enhanced visual function, contribute to holistic well-ness. Research by Kim et al. (2022) has demonstrated significant enhancements in both objective and subjective measures of visual function following upper eyelid blepharoplasty, further emphasizing its functional benefits beyond mere cosmetic enhancement [2]. The procedure’s social and professional benefits, including positive first impressions and career opportunities, further highlight its significance. Papadopulos et al. (2019) and Domela Nieuwenhuis et al. (2022) have both reported on the enhanced social confidence and perceived attractiveness experienced by individuals undergoing upper eyelid blepharoplasty, underscoring its broader impact on social interactions and professional success [3,4]. Long-term satisfaction, a hallmark of this procedure, underscores its transformative nature. Comprehensive assessments using validated instruments such as the FACE-Q questionnaire have consistently shown sustained improvements in patient satisfaction and well-being over time [5].

Our study aims to systematically assess the comprehensive impact of upper eyelid blepharoplasty on patients’ quality of life, employing rigorous methodologies and standardized assessment protocols. By evaluating physical, functional, emotional, and social dimensions, we aim to provide valuable insights to both scientific discourse and clinical practice, enhancing understanding of its holistic benefits and emphasizing the importance of patient-centered care. Through meticulous patient selection and robust outcome evaluation, we seek to consolidate upper eyelid blepharoplasty’s position as a safe, effective intervention for addressing both cosmetic and functional concerns, ultimately striving to optimize patient outcomes and satisfaction.

## 2. Materials and Methods

A prospective, randomized controlled trial was conducted between September 2020 and January 2022. The study protocol received approval from the Institutional Review Board, and written informed consent was obtained from all participants. The study adhered to ethical principles outlined in the Declaration of Helsinki, with participant confidentiality and privacy maintained throughout the study.

Patients aged between 49 and 87 years who underwent upper eyelid surgery during the specified period were considered for inclusion. Exclusion criteria included a history of ocular or orbital trauma, previous eyelid or eyebrow surgery, other cosmetic procedures, ophthalmic disease, and blepharoptosis. Additionally, sequelae after facial paresis were excluded. Eligible participants were randomly assigned to one of two treatment groups: Group A (*n* = 168) received continuous sutures, while Group B (*n* = 180) received intradermal sutures. A standard surgical technique for upper eyelid skin removal was performed on all participants. This involved aseptic skin disinfection using Povidone iodine, local anesthesia with Lidocaine 2% (Lidocaine^®^, Sopharma, Sofia, Bulgaria) + 0.5% bupivacaine (Marcaine^®^, Recipharm Monts, Monts, France) 1:1, assessment of skin to be excised using the pinch technique, scalpel incision, bipolar cautery, and closure with 6-0 Nylon sutures. Steri strips were applied until suture removal to maintain patient blinding. Participants were instructed to avoid heavy lifting, sudden bending, and strenuous sporting activities for 7 days following surgery. Suture strips and sutures were removed during a follow-up visit 7 days postoperatively, with subsequent evaluations at 3, 6, and 12 months to assess for complications. Baseline data were collected before surgery, and follow-up evaluations were conducted at 3, 6, and 12 months postoperatively. Validated FACE-Q questionnaires were used to assess various outcomes including early-life impact, expectations, satisfaction with eyes, satisfaction with overall facial appearance, satisfaction with the outcome, psychological function, social function, and adverse effects.

### Statistical Analysis

The statistical analysis was conducted using SPSS version 26.0 (IBM, Armonk, NY, USA). Patient demographics were delineated, and ratings on the FACE-Q scale, initially ranging from 0 to 4, were transformed into RASCH scores ranging from 0 (worst) to 100 (best). To assess differences between groups, nonparametric tests, including the Friedman test and the Mann–Whitney U test, were employed. After conducting the Friedman test, multiple post hoc pairwise comparison tests were performed to identify specific differences. All *p* values in the results section represent adjusted *p* values following Bonferroni correction. A significance threshold of *p* < 0.05 denoted statistical significance. The results are presented as means with the standard deviation (SD) and median with the interquartile range (IQR).

## 3. Results

Among the original group of 357 participants, 348 completed the study as outlined in the protocol. A total of 9 participants were lost to follow-up after their sutures were removed, leading to their exclusion from the final analysis, which therefore focused on the remaining 348 participants. This group comprised 215 female participants (61.78%) and 133 male participants (38.22%). Additional demographic information is detailed in Table 1.

### 3.1. FACE-Q Assessment (Early-Life Impact, Expectations, Satisfaction with Eyes, Overall Facial Satisfaction, Satisfaction with the Outcome, Psychological and Social Function)

The results indicate that early-life impact significantly increased at all three time points compared to the baseline, regardless of the suturing technique employed (*p* < 0.001). Significantly higher scores were observed in parameters such as expectations, satisfaction with eyes, overall satisfaction, and satisfaction with the outcome at 3 and 6 months postoperatively compared to the baseline (*p* < 0.001) (Table 2). Psychological and social functions were enhanced at 3 months postoperatively compared to the baseline in both groups (*p* < 0.001). However, significant differences between groups were not observed (*p* > 0.05) (Table 2). Across all results, higher RASCH scores denote better outcomes.

### 3.2. Adverse Effects

At the 3-month follow-up, scar visibility following upper eyelid blepharoplasty was minimal for the majority of patients, with 267 individuals reporting no obvious scarring (Table 3). Slight visibility of scars was reported by 69 patients, while only 12 patients rated the visibility as moderate. Symptoms such as dry eye, eye irritation, excessive tearing, and difficulty closing eyes were reported as moderate by 12 patients at this time point (Table 3). By the 12-month follow-up, a significant improvement in scar visibility was observed, with only 4 patients reporting moderately visible scars, and 310 patients reporting no visible scars. Despite these improvements, symptoms such as dry eye, excessive tearing, and difficulty closing eyes persisted in eight patients at the 12-month mark (Table 3).

## 4. Discussion

Upper eyelid blepharoplasty extends beyond superficial alterations, carrying pro-found implications for individuals’ well-being [3]. Facial appearance plays a significant role in shaping one’s sense of happiness and self-esteem, as the face serves as the primary medium through which individuals express emotions, interact socially, and establish their identity. Alterations to facial features, especially those around the eyes, can profoundly impact how individuals perceive themselves and are perceived by others. Facial aesthetics are intricately linked to psychological well-being, with studies consistently demonstrating the influence of facial appearance on self-esteem, confidence, and overall quality of life. It represents more than just a cosmetic procedure; it is a means of restoring harmony to facial features, alleviating insecurities, and empowering individuals to present their best selves to the world [3,6]. By addressing concerns such as sagging eyelids, puffiness, and wrinkles, blepharoplasty can rejuvenate appearance and instill renewed confidence and vitality in patients. Moreover, the eyes are often regarded as the “windows to the soul”, serving as a focal point of interpersonal communication and emotional expression. Alterations in the appearance of the upper eyelids can affect how individuals perceive themselves and are perceived by others, potentially impacting their social interactions, relationships, and overall sense of connection [6].

Research has shown that individuals satisfied with their facial appearance tend to report higher levels of happiness and life satisfaction [7]. Conversely, dissatisfaction with facial features, such as excess skin and fat around the upper eyelids, can lead to feelings of self-consciousness, social withdrawal, and diminished self-worth. Moreover, studies have indicated that dissatisfaction with facial features can extend beyond psychological implications to affect various aspects of individuals’ lives. For example, research by Little et al. (2011) demonstrated that individuals unhappy with their facial appearance may experience limitations in social interactions, such as avoiding social gatherings or feeling uncomfortable in public settings. Additionally, dissatisfaction with facial aesthetics has been linked to decreased confidence in professional settings, potentially hindering career advancement opportunities [7,8]. Furthermore, the impact of facial dissatisfaction can extend to broader aspects of well-being, including mental health. Several studies have highlighted the correlation between dissatisfaction with facial features and symptoms of anxiety and depression [9,10]. Individuals who perceive their facial appearance negatively may experience heightened levels of psychological distress, impacting their overall quality of life and functioning. In the context of upper eyelid blepharoplasty, addressing concerns related to excess skin and fat can alleviate these negative psychological effects. By enhancing facial aesthetics and restoring harmony to the upper eyelids, blepharoplasty can contribute to improved self-esteem, social confidence, and overall well-being [6]. This underscores the importance of cosmetic procedures such as blepharoplasty in not only addressing physical concerns but also promoting mental and emotional health.

The decision to undergo upper eyelid blepharoplasty is often driven by a desire for self-improvement and personal fulfillment [3,4]. For many individuals, addressing concerns about their facial appearance is a deeply personal decision rooted in a desire to align their outward appearance with their inner sense of identity and well-being. By undergoing blepharoplasty, patients reclaim agency over their appearance, asserting control over aspects of their physicality that may have previously caused distress or discomfort. In essence, upper eyelid blepharoplasty transcends its cosmetic implications to become a vehicle for holistic self-enhancement and empowerment [4,6]. By addressing concerns related to facial aesthetics, this procedure enables individuals to project confidence, authenticity, and happiness, ultimately contributing to their overall sense of well-being and fulfillment in life [6]. Thus, the significance of upper eyelid blepharoplasty extends far beyond its physical outcomes, encompassing profound psychological and social dimensions that enrich patients’ lives and promote their happiness and self-actualization.

Several seminal studies have delved into the outcomes of upper eyelid blepharoplasty, consistently highlighting its positive impact on patients’ self-perceptions and overall quality of life. For example, Hollander et al. (2019) conducted a systematic review that encompassed a wide array of patient-reported outcomes following upper eyelid blepharoplasty [11]. Their comprehensive analysis revealed significant improvements across various domains of self-perception and quality of life, including enhanced self-esteem and overall satisfaction among patients. Similarly, Kim et al. (2021) conducted a prospective study focusing on patients’ subjective experiences and satisfaction levels following upper eyelid blepharoplasty [12]. In a retrospective cohort study, Papadopulos et al. (2019) examined the long-term psychological impact of upper eyelid blepharoplasty on patients’ well-being [3]. Their findings revealed sustained improvements in self-esteem, body image, and overall life satisfaction among participants up to five years postoperatively. These results highlight the enduring positive effects of blepharoplasty on patients’ psychological health and happiness. Aladwan et al. (2023) conducted a cross-sectional study investigating the relationship between facial cosmetic procedures, and happiness levels among middle-aged adults [13]. Their findings indicated a significant association between undergoing cosmetic procedures and higher self-reported levels of happiness and life satisfaction. Kim et al. (2021) conducted a prospective cohort study evaluating the effects of upper eyelid blepharoplasty on quality of life and happiness among elderly patients [12]. Their findings revealed significant improvements in physical functioning, emotional well-being, and social interactions following surgery. Participants reported feeling happier and more fulfilled, attributing these positive changes to the aesthetic improvements achieved through blepharoplasty. Through qualitative assessments and patient interviews, they elucidated the nuanced emotional and psychological dimensions of the procedure, highlighting the profound sense of empowerment and self-assurance reported by participants [12]. Furthermore, research has underscored the functional aspects of upper eyelid blepharoplasty, particularly its role in improving visual function and alleviating functional impairment caused by sagging upper eyelids. An et al. (2014) conducted a comprehensive evaluation of changes in visual function following upper eyelid blepharoplasty, demonstrating significant improvements in both objective and subjective measures of visual function postoperatively [14]. These functional enhancements underscore the importance of upper eyelid blepharoplasty in promoting not only aesthetic but also functional well-being.

The choice of suturing technique in upper eyelid blepharoplasty has been a subject of interest in previous research. While the literature lacks consensus on the superiority of one technique over the other, several studies have compared outcomes between continuous, intradermal, and interrupted sutures. For example, a prospective cross-sectional study by Aydemir et al. (2022) found that the interrupted suture technique has lower rates of edema, ecchymosis, and scar formation than the running suture technique [15]. On the other hand, a meta-analysis by Liu et al. (2023) concluded that both continuous and intradermal sutures yield satisfactory aesthetic results with comparable complication rates [16]. Told et al. (2023) conducted a prospective randomized controlled trial comparing outcomes between continuous and interrupted sutures in upper eyelid blepharoplasty [17]. Their study found no significant differences between the two suturing techniques in postoperative complications, wound healing, or aesthetic outcomes. These findings suggest that continuous and interrupted sutures can achieve favorable outcomes in upper eyelid blepharoplasty. Baek et al. (2015) compared outcomes between continuous and interrupted buried knot sutures in upper eyelid blepharoplasty [18]. Their analysis revealed that the continuous buried suture method has fewer complications than the interrupted one. Rodrigues et al. (2023) conducted a systematic review and meta-analysis to assess different surgical techniques and their outcomes in upper eyelid blepharoplasty [19]. The review found a small number of complications reported and equal patient satisfaction with the aesthetic outcomes following upper eyelid blepharoplasty, regardless of the surgical technique used.

The FACE-Q questionnaire stands as a cornerstone in the evaluation of patient-reported outcomes in facial aesthetic procedures, including upper eyelid blepharoplasty. Its extensive validation and demonstrated reliability make it a robust tool for assessing various dimensions of patient satisfaction and well-being. Studies have consistently shown high internal consistency and test–retest reliability, affirming its efficacy in capturing the nuanced impact of surgical interventions on patients’ lives [20]. What sets the FACE-Q questionnaire apart is its comprehensive assessment of diverse domains relevant to facial aesthetic procedures, offering insights beyond mere physical outcomes. It delves into aspects such as satisfaction with appearance, psychosocial well-being, quality of life, and patient expectations. This holistic approach provides a nuanced understanding of the multifaceted effects of procedures like upper eyelid blepharoplasty, shedding light on both the tangible and intangible outcomes that influence patients’ overall satisfaction [17]. In clinical practice, the FACE-Q questionnaire serves as a valuable tool for healthcare providers to systematically assess patients’ satisfaction and progress throughout the treatment journey. By integrating patient-reported outcomes into clinical decision making, providers can identify areas of concern, address patient expectations, and tailor treatment plans to align with individual needs. This personalized approach not only enhances treatment outcomes but also fosters a stronger patient–provider relationship built on trust and communication [17,20]. Moreover, in the realm of research, the FACE-Q questionnaire plays a pivotal role in evaluating the effectiveness of facial aesthetic procedures, including upper eyelid blepharoplasty. Its widespread utilization in clinical studies facilitates rigorous evaluation of treatment efficacy and longitudinal follow-up, providing valuable insights into the long-term impact of surgical interventions on patient well-being [17]. Furthermore, the FACE-Q questionnaire enables comparative studies, allowing researchers to objectively assess treatment outcomes across different surgical techniques, interventions, or patient populations. By standardizing assessment tools across studies, researchers can conduct meta-analyses and evidence-based decision making, advancing the field of facial aesthetic surgery and informing best practices in patient care [21].

In light of these previous findings and our own results, it is evident that the choice of suturing technique may not substantially impact patients’ satisfaction with the outcomes of upper eyelid blepharoplasty. This underscores the importance of individualized treatment planning and patient-centered care, where factors such as patient preferences, anatomical considerations, and surgeon expertise should guide the selection of suturing techniques. Furthermore, our study utilized FACE-Q questionnaires to assess patients’ overall happiness and satisfaction following upper eyelid blepharoplasty. The findings of this study demonstrate significant improvements in various aspects of patients’ well-being following upper eyelid blepharoplasty, regardless of the suturing technique employed. Firstly, patients experienced a notable enhancement in their contentment with their expectations, overall facial appearance, satisfaction with the outcome, psychological function, and social function compared to baseline (*p* < 0.05). These improvements suggest that upper eyelid blepharoplasty not only addresses physical concerns but also positively impacts patients’ psychological and social well-being. The significant increase in satisfaction with eyes suggests that patients were highly content with the aesthetic outcomes of the procedure, with the majority reporting minimal to no visible scarring at the 3-month follow-up. This improvement in scar visibility further progressed over time, with a substantial reduction in moderately visible scars by the 12-month mark. Despite this improvement, some patients experienced persistent symptoms such as dry eye, excessive tearing, and difficulty closing eyes, highlighting the importance of postoperative care and the management of potential adverse effects. The absence of significant differences between suturing techniques regarding various outcome measures indicates that both continuous and interrupted sutures yield comparable results in terms of patient satisfaction and functional outcomes. This finding is consistent with previous research suggesting that the choice of suturing technique may not substantially impact surgical outcomes in upper eyelid blepharoplasty. Moreover, the consistent improvements in patients’ happiness and satisfaction over time highlight the enduring positive impact of the procedure on their quality of life. As such, our study emphasizes the importance of utilizing patient-reported outcome measures, such as the FACE-Q questionnaire, to comprehensively evaluate the effectiveness of surgical interventions and tailor treatment approaches to meet individual patient needs and preferences. Our study’s findings highlight the importance of incorporating the FACE-Q questionnaire into clinical practice. By utilizing this validated tool, healthcare providers can systematically evaluate various dimensions of patient satisfaction and well-being, enabling a more comprehensive understanding of the impact of upper eyelid blepharoplasty on patients’ lives. Through regular assessments using the FACE-Q questionnaire, providers can identify areas of concern, address patient expectations, and optimize treatment strategies to ensure optimal outcomes and patient-centered care. By leveraging this standardized assessment tool, healthcare providers can enhance patient-centered care, optimize treatment outcomes, and ultimately improve the overall patient experience. However, further research is warranted to explore long-term outcomes and potential factors influencing patient satisfaction following upper eyelid blepharoplasty.

## 5. Conclusions

In summary, our study underscores the beneficial effects of upper eyelid blepharoplasty on patients’ quality of life, aligning with a growing body of evidence. By consolidating existing research and offering fresh insights, we highlight the necessity of employing a thorough approach to assessing the results of cosmetic procedures. As cosmetic surgery progresses, ongoing research and standardized evaluation methods remain crucial for advancing patient care and refining treatment outcomes.

## Figures and Tables

**Table 1:** Demographical characteristics. Group A: continuous suture. Group B: intradermal suture.

Groups	Gender	Age (Years) Mean ± SD
Group A (*n* = 168)	Male (*n* = 69) 41.07%	73.22 ± 9.66
	Female (*n* = 99) 58.93%	69.16 ± 11.51
Group B (*n* = 180)	Male (*n* = 64) 35.6%	73.77 ± 10.61
	Female (*n* = 116) 64.4%	70.19 ± 11.21

**Table 2:** Comparison of median (Q1; Q3) pre-operative, 3 months, 6 months, and 12 months postoperative FACE-Q scores.

FACE-Q Assessment	Early-Life Impact	Expectations	Satisfaction with Eyes	Overall Face Satisfaction	Satisfaction with the Outcome	Psychological Function	Social Function
Group A							
Preoperative	40 (30; 43)	32 (23; 35)	35 (20; 35)	33 (31; 33)	28 (24; 31)	40 (23; 42)	41 (35; 44)
3 months postoperative	66 (58; 70)	65 (62; 77)	68 (59; 72)	66 (55; 69)	73 (73; 79)	77 (77; 84)	66 (66; 73)
	*p* < 0.001 *	*p* < 0.001 *	*p* < 0.001 *	*p* < 0.001 *	*p* < 0.001 *	*p* < 0.001 *	*p* < 0.001 *
6 months postoperative	73 (66; 73)	77 (77; 83)	72 (59; 72)	69 (66; 72)	79 (73; 87)	77 (77; 84)	66 (66; 73)
	*p* < 0.001 *,**	*p* < 0.001 *,**	*p* < 0.001 *,**	*p* < 0.001 *,**	*p* = 0.041 *,**	*p* > 0.05	*p* > 0.05
12 months postoperative	77 (70; 82)	77 (62; 83)	72 (59; 72)	66 (66; 72)	79 (73; 87)	84 (77; 84)	66 (66; 73)
	*p* < 0.001 *,***	*p* > 0.05	*p* = 0.067	*p* > 0.05	*p* > 0.05	*p* > 0.05	*p* > 0.05
Group B							
Preoperative	40 (37; 43)	35 (23; 35)	35 (20; 39)	33 (28; 33)	28 (24; 35)	42 (23; 42)	38 (35; 44)
3 months postoperative	61 (58; 70)	69 (62; 77)	63 (59; 72)	66 (64; 69)	73 (68; 79)	77 (71; 84)	66 (62; 70)
	*p* < 0.001 *	*p* < 0.001 *	*p* < 0.001 *	*p* < 0.001 *	*p* < 0.001 *	*p* < 0.001 *	*p* < 0.001 *
6 months postoperative	70 (58; 73)	77 (73; 83)	72 (59; 77)	66 (66; 72)	79 (73; 87)	80 (77; 84)	66 (66; 73)
	*p* = 0.001 *,**	*p* < 0.001 *,**	*p* < 0.001 *,**	*p* < 0.001 *,**	*p* < 0.001 *,**	*p* > 0.05	*p* > 0.05
12 months postoperative	73 (70; 90)	77 (73; 83)	68 (59; 72)	69 (66; 72)	87 (73; 87)	84 (77; 88)	73 (66; 73)
	*p* = 0.001 *,***	*p* > 0.05	*p* = 0.029 ***	*p* = 0.045 ***	*p* > 0.05	*p* = 0.020 ***	*p* > 0.05

* *p* < 0.05 vs. preoperative, ** *p* < 0.05 vs. 3 months postoperative and *** *p* < 0.05 vs. 6 months postoperative. Group A: continuous suture. Group B: intradermal suture.

**Table 3:** Adverse effects at 3 and 12 months postoperatively. Group A: continuous suture. Group B: intradermal suture.

3 Months Postoperatively	Groups	Not at All	A Little	Moderate
Eyelid scar visibility	Group A (*n* = 168)	134 (79.76%)	30 (17.86%)	4 (2.38%)
	Group B (*n* = 180)	133 (73.89%)	39 (21.67%)	8 (4.44%)
Eye dryness	Group A (*n* = 168)	144 (85.71%)	17 (10.20%)	7 (4.17%)
	Group B (*n* = 180)	154 (85.56%)	21 (11.67%)	5 (2.78%)
Irritation of the eye	Group A (*n* = 168)	136 (80.95%)	26 (15.48%)	6 (3.57%)
	Group B (*n* = 180)	150 (83.33%)	24 (13.33%)	6 (3.33%)
Excessive tearing	Group A (*n* = 168)	145 (86.31%)	15 (8.93%)	8 (4.76%)
	Group B (*n* = 180)	158 (87.78%)	18 (10.00%)	4 (2.22%)
Difficulty closing eyes	Group A (*n* = 168)	153 (91.07%)	10 (5.95%)	5 (2.98%)
	Group B (*n* = 180)	166 (92.22%)	7 (3.89%)	7 (3.89%)
12 months postoperatively		Not at all	A little	Moderate
Eyelid scar visibility	Group A (*n* = 168)	149 (88.69%)	16 (9.52%)	3 (1.79%)
	Group B (*n* = 180)	161 (89.44%)	18 (10.00%)	1 (0.56%)
Eye dryness	Group A (*n* = 168)	148 (88.10%)	17 (10.12%)	3 (1.78%)
	Group B (*n* = 180)	160 (88.88%)	15 (8.34%)	5 (2.78%)
Irritation of the eye	Group A (*n* = 168)	149 (88.69%)	15 (8.93%)	4 (2.38%)
	Group B (*n* = 180)	165 (91.67%)	11 (6.11%)	4 (2.22%)
Excessive tearing	Group A (*n* = 168)	149 (88.69%)	15 (8.93%)	4 (2.38%)
	Group B (*n* = 180)	169 (93.89%)	7 (3.89%)	4 (2.22%)
Difficulty closing eyes	Group A (*n* = 168)	157 (93.45%)	6 (3.57%)	5 (2.98%)
	Group B (*n* = 180)	171 (95.00%)	6 (3.33%)	3 (1.67%)

## Data Availability

Data is contained within the article.
